# “CARING in Action”: A Knowledge Mobilization Project on Co-Creating Evidence-Based Communication Training in Chronic Pain Care for Healthcare Professionals

**DOI:** 10.1080/24740527.2026.2696249

**Published:** 2026-08-06

**Authors:** Doriana Taccardi, Nataly R. Espinoza-Suarez, Swapnil Shah, Jennifer Daly-Cyr, Annie LeBlanc, Nader Ghasemlou, Lynn Cooper, Rachael Bosma, Rachel Roy

**Affiliations:** a Department of Biomedical & Molecular Sciences, Queen’s University, Kingston, Ontario, Canada; b VITAM Research Center on Sustainable Health, Quebec Integrated University Health and Social Services Center, Québec City, Québec, Canada; c Chronic Pain Network, McMaster University, Hamilton, Ontario, Canada; d Knowledge Evaluation Research Unit, Mayo Clinic, Rochester, Minnesota, USA; e Faculty of Medicine, Laval University, Québec City, Québec, Canada; f Department of Anaesthesiology & Perioperative Medicine, Queen’s University, Kingston, Ontario, Canada; g Krembil Brain Institute, University Health Network, Toronto, Ontario, Canada; h Department of Immunology, University of Toronto, Toronto, Ontario, Canada; i Centre for the Study of Pain, University of Toronto, Toronto, Ontario, Canada; j Toronto Academic Pain Medicine Institute, Women’s College Hospital, Toronto, Ontario, Canada

**Keywords:** Knowledge mobilization, communication, chronic pain, pain care, healthcare, patient-oriented research

## Abstract

**Background:**

Pain is a complex and multifactorial experience shaped by biopsychosocial and contextual factors. Communication gaps between healthcare professionals and people living with pain persist as a major barrier to effective and patient-centered care. Supporting the education of healthcare professionals is essential to improving patient satisfaction and the overall experience of care for both healthcare professionals and people living with pain.

**Aims:**

To address this gap in pain care communication, this paper aims to describe the *CARING in Action* project, a patient-oriented approach to co-developing resource materials and a dissemination strategy to enhance knowledge mobilization and implementation.

**Methods:**

This project employed a participatory co-creation design informed by Canada’s Strategy for Patient-Oriented Research. Patient partners contributed across all stages, from idea conceptualization, to planning and dissemination. We identified communication gaps, adapted evidence to context, and held iterative virtual and in-person workshops with people with lived experience, healthcare professionals, researchers, and trainees. Prototype materials were refined through three cycles of iteration, resulting in an evidence-informed, educational tool shaped by diverse interest holder perspectives and strengthened by a process prioritizing consensus-building, inclusivity, and validation.

**Results:**

The final eight-module is a self-directed online educational resource for licensed and trainee healthcare professionals, to strengthen effective communications with people living with pain.

**Discussion:**

This initiative generated concrete knowledge products that support culturally attuned communication and relational approaches to pain management. Together, these outputs provide practical resources for healthcare professionals and trainees across pre-licensure and post-licensure education, supporting the implementation of patient-centered communication practices in pain care.

## Résumé

**Contexte :** La douleur est une expérience complexe et multifactorielle, façonnée par des facteurs biopsychosociaux et contextuels. Les lacunes dans la communication entre les professionnels de la santé et les personnes vivant avec la douleur persistent et constituent un obstacle majeur à l’efficacité des soins et à la prestation de soins centrés sur le patient. Il est essentiel de soutenir la formation des professionnels de la santé afin d’améliorer la satisfaction des patients ainsi que l’expérience de prise en charge, tant pour les professionnels de la santé que pour les personnes vivant avec la douleur.

**Objectifs :** Pour remédier à cette lacune dans la communication en matière de soins liés à la douleur, cet article vise à décrire le projet CARING in Action, une approche axée sur le patient qui consiste à concevoir, avec les personnes concernées, des ressources et une stratégie de diffusion visant à favoriser la mobilisation et l’application des connaissances.

**Méthodes :** Ce projet reposait sur une démarche participative de cocréation inspirée de la Stratégie de recherche axée sur le patient du Canada. Les patients partenaires ont contribué à toutes les étapes du projet, de la conceptualisation de l’idée à la planification et à la diffusion. Nous avons cerné les lacunes en matière de communication, adapté les données probantes au contexte et tenu une série d’ateliers itératifs, en mode virtuel et en personne, réunissant des personnes ayant une expérience vécue, des professionnels de la santé, des chercheurs et des personnes en formation. Les prototypes ont été affinés au cours de trois cycles d’itération, ce qui a mené à la création d’un outil éducatif éclairé par les données probantes, façonné par les perspectives de diverses parties prenantes et renforcé par une démarche accordant la priorité à la recherche de consensus, à l’inclusivité et à la validation.

**Résultats :** La ressource finale, composée de huit modules, est un outil éducatif en ligne que les professionnels de la santé titulaires d’un permis d’exercice ou en formation peuvent suivre de façon autonome afin de communiquer plus efficacement avec les personnes vivant avec la douleur.

**Discussion :** Cette initiative a permis de produire des outils concrets de mobilisation des connaissances qui favorisent une communication sensible aux réalités culturelles ainsi que des approches relationnelles de la prise en charge de la douleur. Ensemble, ces outils offrent des ressources pratiques aux professionnels de la santé et aux personnes en formation, tant avant qu’après l’obtention de leur permis d’exercice, et favorisent la mise en œuvre de pratiques de communication centrées sur le patient dans la prise en charge de la douleur.

## Introduction

Non-communicable diseases, like chronic pain, are among the greatest contributors to disability worldwide.^1^ More than half of Canadians seeking health care present with pain,^2,3^ yet pain remains “everyone’s and no one’s problem” in clinical practice. Although people living with pain receive care across multiple disciplines and care settings, pain management often requires coordination across providers, which can contribute to fragmented care and challenges in establishing clear responsibility for ongoing support.^4,5^ Effective pain care requires a truly multidisciplinary approach in which healthcare professionals, including physicians, nurses, psychologists, physiotherapists, pharmacists, and more, collaborate to provide comprehensive and interdisciplinary care.^6,7^ Strengthening education on interprofessional pain management, clarifying scopes of practice, and defining shared roles are essential for reducing fragmented care and promoting effective teamwork.^8–10^ Chronic pain, in particular, is one of the most common concerns in primary care, yet both healthcare professionals and patients consistently report dissatisfaction with training in pain management and with the quality of pain care itself.^11,12^ For the one in five Canadians living with chronic pain,^2,3^ the first point of contact is typically a primary care professional, who is often insufficiently trained in pain assessment and management.^13^ These early encounters significantly shape patients’ care trajectories and influence long-term outcomes. Identifying and addressing pain, or the risk of chronic pain, early in the care journey can reduce individual suffering and lower long-term healthcare costs.^2^

Those living with chronic pain often feel that the realities of how pain affects their lives and care experiences are not easily communicated or adequately recognized by healthcare professionals.^14–16^ The recently revised definition of pain by the International Association for the Study of Pain as “an unpleasant sensory and emotional experience associated with, or resembling that associated with, actual or potential tissue damage” highlights the subjective nature of pain.^17^ Pain’s invisible and deeply subjective nature challenges the traditional paradigms that rely on objective indicators.^18^ Because pain is ultimately defined by what individuals report, effective care hinges on communication: patients need to find ways to express their experiences, while healthcare professionals should be equipped to ask meaningful questions, validate patients’ perspectives, support adaptive reappraisal when appropriate, and interpret these accounts through a biopsychosocial lens.^19–23^ Indeed, communication may also influence how individuals make sense of and respond to pain, shaping coping processes, self-efficacy, and treatment engagement through supportive reappraisal and shared understanding.^24,25^

Overall, communication represents a persistent barrier to delivering effective care for chronic pain.^26,27^ This communication challenge becomes even more pronounced for women, immigrants, and people with lower socioeconomic status, who often encounter systemic inequities that restrict access to appropriate pain care.^28–30^ Despite extensive research highlights the biopsychosocial dimensions of pain and the central role of therapeutic relationships,^31–34^ many people living with chronic pain continue to face interactions shaped by misunderstanding, minimization, or disbelief.^35–38^ Such experiences reveal complex structural and cultural barriers within healthcare settings, where subjective accounts are frequently overshadowed by biomedical expectations for measurable evidence.^39–41^ These dynamics reinforce stigma and inequities and ultimately perpetuate injustice in pain care.^42^ Strengthening communication and foregrounding the lived perspectives of individuals with pain are therefore essential to delivering equitable, responsive, and patient-centered care.^22^

Although healthcare professionals increasingly recognize the importance of empathetic, patient-centered communication, many report lacking the skills, time, and tools needed for meaningful conversations about pain.^43–48^ Indeed, validation and patient-centered communication may support the initial development of connection and strengthen relational trust and therapeutic alliance by helping patients feel heard, understood, and respected; thereby fostering a sense of safety, partnership, and sustained engagement in care rather than fragmented healthcare experiences.^49,50^ Existing health professional education curricula frequently devote insufficient attention to pain management, particularly with respect to communication competencies,^13,51–56^ with few dedicated modules and minimal instructional time.^57,58^ In parallel, patients often struggle to articulate their pain in ways that are heard and validated, leading to frustration and disengagement on both sides of the clinical encounter.^59–62^

Thus, strengthening the education across the continuum of professional learning – from pre-licensure training to continuing professional development for practicing clinicians – is essential for improving patient satisfaction and enhancing the overall care experience for healthcare professionals and people living with pain.^63^ Interactive and multimodal approaches to continuing professional development are particularly effective in supporting these competencies.^64^ Empathetic, patient-centered communication reduces stigma, supports shared decision-making, and is associated with improved patient-reported outcomes such as satisfaction, quality of life, and communication; fostering greater confidence and engagement in pain self-management and involvement in care decisions.^65^ At the same time, traditional educational resources often fail to reflect the complexity of pain or the realities of clinical encounters. To ensure that training is relevant, actionable, and grounded in lived experience, it is essential to involve patients and their diverse perspectives in the co-creation of educational materials.^66,67^ Patient-oriented research (POR) offers a structured way to embed these perspectives, making educational interventions more impactful, contextually grounded, and aligned with the needs of those most affected. Co-creation allows educators, healthcare professionals, and individuals with lived experience to collectively identify communication challenges, develop a shared language around pain and care experiences, validate content, and design resources that resonate across clinical and educational settings. This collaborative approach is increasingly recognized as a cornerstone of high-quality research and has meaningfully advanced the field of pain science and care by reflecting lived experience voices, supporting their agency, and ensuring that research and educational priorities remain aligned with the needs of those most affected by pain.^68,69^

We created *CARING in Action*, a knowledge mobilization initiative supported by the Chronic Pain Network (CPN) and Canada’s Strategy for Patient-Oriented Research. This initiative grew out of a targeted effort to foster innovation in the pain field and to build knowledge-mobilization capacity among trainees, offering a unique opportunity to co-create patient-oriented educational tools. *CARING in Action* was co-created by a team of individuals with lived experience of chronic pain, healthcare professionals, educators, researchers, and graduate trainees from both clinical and translational science disciplines, providing perspectives from multiple stages of professional and research training. The project takes the form of a set of multimodal training modules, grounded in evidence, that aim to educate healthcare professionals in effective communication strategies for working with people who experience chronic pain. The resulting eight-module, multimodal training resource provides evidence-based guidance to support effective pain communication among learners and healthcare professionals across disciplines, clinical settings, and stages of professional development. Its development directly responds to persistent gaps in pain communication and aligns with national priorities in chronic pain knowledge mobilization, including the recommendations of the Canadian Task Force Report in Health Canada’s *Action Plan for Pain in Canada*.^2,3^

This paper aims to illustrate the co-creation methodology used to develop the *CARING in Action* tool. We outline the participatory processes that guided each stage of the project, from problem identification through content creation and validation, and discuss how this approach supports the development of patient-informed educational resources that address critical gaps in clinical practice. In doing so, we contribute to the growing field of patient-oriented education and highlight the potential of co-created interventions to mobilize knowledge and transform pain care.

## Conceptual framework

This project is informed by participatory research methodologies and aligns with the principles of Canada’s SPOR,^70^ which emphasize meaningful collaboration with people with lived experience across all phases of the research process, including idea conceptualization, project design, literature review, development and refinement of the educational tool, dissemination, and planning. The CARING in Action modules exemplify a commitment to co-creation, fostering equitable engagement with individuals living with pain.

Central to the project’s conceptual foundation is the notion of *situated knowledge*, the understanding that individuals with lived experience possess contextually embedded insights that are vital to the design and delivery of effective educational interventions.^71–73^ By positioning these individuals not as passive recipients but as co-creators of the intervention, the project aims to disrupt traditional hierarchies in health education and research,^74,75^ thereby advancing a participatory model of curriculum development informed by real-world relevance and responsiveness. The content of the *CARING in Action* modules is shaped by established principles of *empathetic and patient-centered communication*,^76^ particularly within the context of chronic pain care. Communication is approached as a modifiable behavior, supported by evidence that effective relational care reduces stigma, enhances therapeutic relationships, and facilitates shared decision-making.^77–79^ These insights inform both the pedagogical structure and the behavioral objectives of this educational tool.

**Figure 1.**
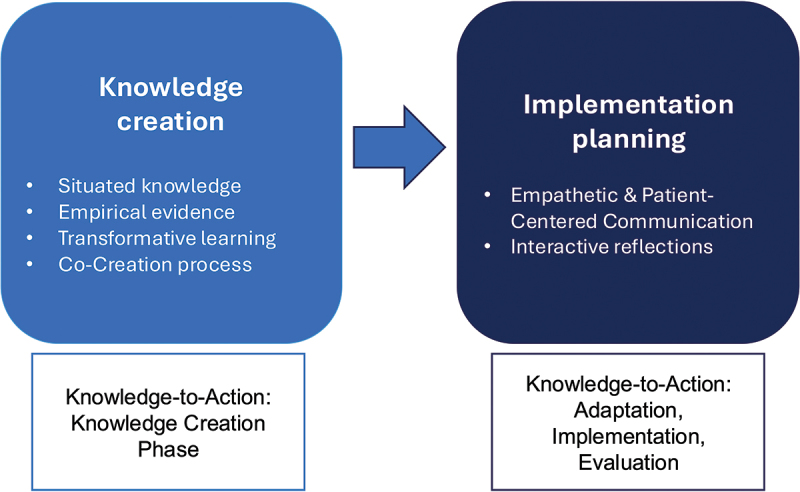
Knowledge mobilization framework diagram.

To guide both creation and implementation, the project adopts the *Knowledge-to-Action* framework,^80^ a widely applied model of knowledge mobilization that conceptualizes a dynamic and iterative process for translating knowledge into practice. The creation of this tool aligns with the knowledge creation phase, integrating experiential (patient-derived) and empirical (research-based) evidence. In addition, the design of the eight-module blended text, video, and interactive reflections was informed by best practices in health professional education and grounded in principles of *transformative learning theory*.^81,82^ Transformative learning benefits the project by ensuring that the modules are not only informative but also transformative in shaping mind-sets, attitudes, and practices sustainably. Our audience analysis led us to adopt a pedagogical approach that encourages critical reflection, challenges assumptions, and supports meaningful changes in practice. Collectively, these integrated conceptual foundations support a robust, coherent theory of change that links co-creation, patient engagement, and the development of communication competencies with broader goals in knowledge implementation and healthcare improvement (See Figure 1).

Overall, the conceptual foundations described in this section informed both the development process and the structure of the CARING in Action tool. The following sections describe the participatory co-creation process used to develop the educational resource, including interest holder engagement, content development, iterative refinement, and knowledge mobilization activities. Then, we present the resulting training resource and associated knowledge products generated through this process.

## Methodology of the co-creation process

Considering the aim of this manuscript is to describe the co-creation and development of an educational resource, the following sections provide an overview of the steps used throughout the project. Firstly, we describe the co-creation methodology that informed resource development, followed by a description of the resulting educational tool and associated knowledge mobilization outputs (see Supplemental Figure 1 for a visual of methodology and workflow).

As this project focused on the co-creation and development of educational resources as a knowledge mobilization initiative rather than the collection of research data for research purposes, research ethics board approval was not required.

Patient engagement and co-creation activities were reported in accordance with the Guidance for Reporting Involvement of Patients and the Public (GRIPP2) short-form reporting guideline,^83^ with the completed checklist provided in Supplemental Table 1.

### Process development

The co-creation of *CARING in Action* emerged from a collaborative project hosted by the Chronic Pain Network’s Training and Capacity Building Committee designed to foster innovation in pain education through rapid, team-based co-creation. This innovative model took place from August 2024 to May 2025 via a hackathon project that brought together approximately 25 participants, including people with lived and living experience (PWLLEs) of chronic pain, graduate student trainees, clinical professionals, knowledge mobilization specialists, and pain researchers, to form diverse and interprofessional teams. Participants were identified through the Chronic Pain Network and were compensated with either professional development awards (trainees) or with honorarium (PWLLEs). These teams were tasked with “hacking” priority issues identified through CPN’s patient-centered priority setting initiatives and further refined by the network’s Knowledge Mobilization and Implementation Science, Patient Engagement, and Equity, Diversity and Inclusion committees. The CARING in Action resource was subsequently developed by a core co-creation team comprising two graduate trainees from clinical and translational science disciplines, three researchers with expertise in clinical and preclinical pain research, three PWLLEs, and one knowledge broker (*n* = 9).

Rooted in knowledge mobilization and co-creation, this interdisciplinary, cross-sectoral, and lived experience-informed collaboration aimed to propose concrete solutions to persistent challenges in pain care, specifically, the ongoing communication gap between healthcare professionals and people living with pain. This event provided a structured environment for participants to collaboratively design and prototype educational tools, embodying the principles of participatory research and knowledge co-production central to Canada’s SPOR.

### Design

The training tool was fully created on Articulate and is hosted online to facilitate broad accessibility. Ongoing maintenance and dissemination are supported through the Chronic Pain Network’s knowledge mobilization activities. The process was structured into several key stages. First, participants engaged in collaborative brainstorming to identify core communication problems based on both clinical insights and lived experiences (approximately 1–2 months). Next, we identified the key audience for the content based on the issues we landed on as priorities and assessed different knowledge mobilization tactics to determine that a training module interface would be best suited to this identified problem (approximately 1–2 months). This was followed by a prioritization phase, in which proposed topics were discussed during team meetings and ranked through facilitated group discussion and voting. Final priorities were selected through consensus among the core co-creation team, ensuring that both lived experience and professional perspectives were represented in decision-making. Then, teams worked collaboratively to create the core content and guiding principles, ensuring both selection of evidence-based content and alignment with patient-centered care values (approximately 3–4 months). Content development was informed by a non-exhaustive review of peer-reviewed and gray literature on pain communication, patient-centered care, validation, and therapeutic relationships, including evidence being synthesized as part of an ongoing scoping review of humanistic communication in pain care. This evidence was supplemented by the clinical, research, knowledge mobilization, and lived-experience expertise represented within the co-creation team. The final stage involved validating key messages, reviewing the tone and content for clarity, and ensuring the use of inclusive and respectful language (approximately 1–2 months). Draft graphics and modules were circulated to healthcare professionals, researchers, and PWLLEs within the CPN network for review. Feedback was collected through structured discussions, written comments, and iterative revisions (three main in total) focusing on content accuracy, clarity, tone, accessibility, and relevance to clinical practice. Revisions were incorporated and re-reviewed until consensus was achieved within the core co-creation team. From the outset, we integrated feedback from PWLLEs within the CPN-SPOR network, including individuals who self-identify as members of 2SLGBTQ2I+ and Indigenous communities, into the initial design of our training module. The validation process ensured that the training content aligned with how collectively we want to see communication on pain improved. This iterative and democratic design process was based on collective decision-making within the team and larger network; fostering shared ownership, creativity, and relevance, laying a strong foundation for a tool that is both practical and informed by the realities of chronic pain care. Finally, it was collectively decided that the content would be provided in both English and French to maximize accessibility across the country.

### Modalities of collaboration

A series of interactive virtual workshops served as the foundation for this collaboration, enabling sustained and flexible engagement across geographic regions and participant roles. Initially held on a monthly basis, these meetings increased in frequency, eventually occurring biweekly and then weekly as the project progressed. This cadence supported the evolving needs of the team and allowed for deeper iterative creation. To support idea generation and knowledge integration, a range of collaborative techniques was employed. These included structured brainstorming, concept mapping to visualize key themes and relationships, and peer review for content validation and critical reflection. Shared documents were used as a tool for collaboration across diverse geolocations. Many of these techniques were employed intensively during a dedicated in-person retreat in October 2024 hosted by CPN-SPOR, which served as a key milestone for advancing the tool’s design.

Decision-making was guided by consensus-building strategies and affinity voting, ensuring transparency, shared ownership, and thoughtful resolution of differing perspectives. Consistent with SPOR principles of equitable interest holder engagement, the project prioritized mutual respect, support, inclusiveness, and co-creation. When divergent viewpoints emerged, facilitated discussions were used to explore underlying concerns, identify common ground, and balance empirical evidence with lived-experience perspectives. The main principles emerged through an iterative co-creation process that integrated evidence from the literature on pain communication, patient-centered care, therapeutic relationships, validation, and chronic pain management with lived-experience perspectives, clinical expertise, and interest holder input. The principles were subsequently refined through group discussions, iterations, and affinity voting, drawing on patient testimonies, clinical scenarios, and communication research to ensure that each component was both conceptually meaningful and practically applicable.

This multimodal, participatory process allowed for creativity, deepened mutual understanding, and helped ensure that the resulting training tool was not only evidence-informed but also meaningfully shaped by the experiences and priorities of those it is intended to serve.

## Results

### Co-creation outcomes: the CARING framework

**Figure 2.**
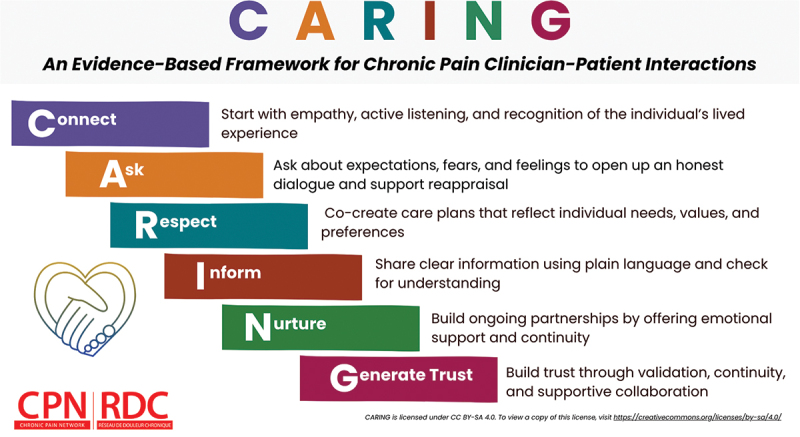
CARING in Action graphic design english version.

**Figure 3.**
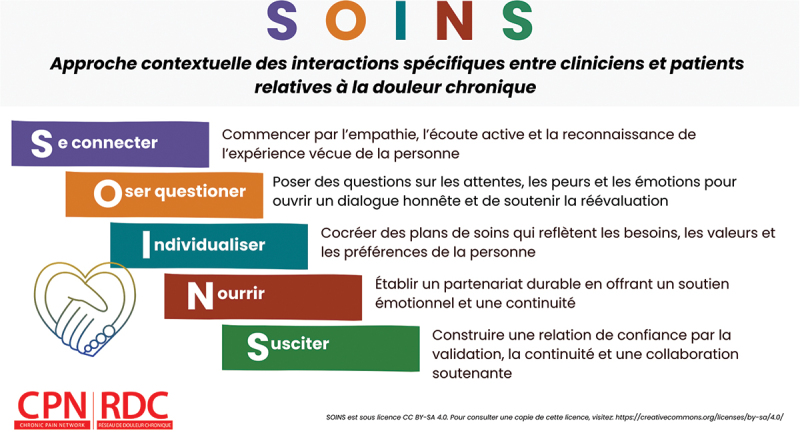
CARING in Action graphic design French version. Le modèle de soins, développé par les membres du Chronic Pain Network, Doriana Taccardi, Nataly Espinoza Suarez, Rachel Roy, Swapnil Shah et Jennifer Daly-Cyr.

The final product of this co-creation process is the *CARING Framework*, a set of educational training modules structured around six key principles, each corresponding to a letter of the acronym *CARING*. These principles are: ***C****onnect* through careful and empathetic conversations; ***A****sk* about understanding, feelings, expectations, and support reappraisal; ***R****espect* each person’s needs and wishes; ***I****nform* in a clear and truthful way; ***N****urture* relationships with companionship and support; and ***G****enerate* trust through continuity and supportive collaboration (Figures 2 and 3). The SOINS framework (Figure 3) represents a French-Canadian linguistic and cultural adaptation of the original CARING framework. This adaptation was developed collaboratively with Francophone collaborators and PWLLEs to enhance conceptual clarity, cultural resonance, usability, and memorability within French-speaking healthcare contexts. The term SOINS was intentionally selected because it closely reflects the relational and care-centered philosophy underlying the concept of “caring” in clinical interactions. The acronym contains five letters, as certain concepts were grouped differently to preserve linguistic coherence and usability in French while maintaining the full conceptual content of the six-step CARING framework.

Each element of the framework emerged through an iterative co-creation process that integrated evidence from the literature on pain communication, patient-centered care, therapeutic relationships, validation, and chronic pain management with lived-experience perspectives, clinical expertise, and stakeholder consensus. Participants included a core group composed of a total of nine members: three individuals with lived experience of pain, two PhD trainees, one knowledge broker, and three chronic pain scientists (both clinical and pre-clinical). This team received advice and expertise from the larger Chronic Pain Network, which is composed of around 114 members in its governance structure, including healthcare professionals, researchers, and people living with pain. The core group emphasized the need for healthcare professionals to move beyond technical communication and toward relational care grounded in empathy, mutual respect, and trust. The CARING/SOINS principles provide a simple yet powerful guide to support healthcare professionals in fostering compassionate, inclusive, and effective communication with people living with chronic pain.

### Training format

The *CARING in Action* Framework was developed as an interactive online educational platform composed of eight modules. It begins with an introduction module where the CARING framework is explained and contextualized. The CARING acronym thereafter unfolds within our online training course, with each letter representing a separate module (six for the English version and five for the French version). There is a consistent structure for each module that includes some basic action points supported by literature, each point gets expanded with additional content (e.g., testimony from people with lived experience, quotes, and insights from healthcare professionals and leaders in the pain space) and resources (e.g. references peer reviewed journals, links to other national organizations, policies). The content is interactive and uses modalities including videos, stories, quotes, to engage the audience. Each module includes a demo video to provide an example of how to apply the actions learned in the module in a real-life interaction.

Each module was designed to be completed in approximately 5–10 minutes for a total time of about 40–60 minutes, with users able to access additional resources according to their interests and learning needs. At the end of each module, users complete a reflection exercise to integrate their learning. The training course concludes with a module where the key takeaways from the training are reiterated, and all the available resources, infographics, and supportive documents can be downloaded. The content is accessed online, can be integrated into the existing healthcare curricula and other online e-learning platforms and is intended for healthcare professionals and trainees across disciplines and stages of professional development, including both pre-licensure learners and practicing clinicians. Additional infographic (Figures. 2 and 3) and one-pager with a summary of key takeaways are included at the end of the training course and can be downloaded in both English and French (see Supplemental figures for screenshots from the training platform).

#### Knowledge mobilization considerations on the training format

The design of the modules was based on knowledge mobilization considerations of practitioner time and capacity. We created them in single, short modules with additional resources linked to them to allow the users to spend as much or as little time as they have available to complete the content. We chose to divide the training into eight separate modules so they could be completed one at a time without having to sit for a longer sustained amount of time. We considered the learning needs of the intended audience and presented the content in a way that frames the skills as enhancing their current practice rather than making any changes. The goal of including the demonstration videos was to show the audience that the concepts do not need to add to their time pressures in clinical interactions; rather, these techniques could be employed within their brief routine interactions. Although the communication principles may have broader applicability, the examples, scenarios, and content were primarily developed within the context of adult chronic pain care.

## Implementation considerations and lessons learned

This project reaffirmed the critical value of co-creating educational tools in partnership with PWLLEs.^84^ Their active engagement brought indispensable depth, authenticity, and contextual relevance to the creation of the *CARING in Action*. Far from occupying tokenistic or consultative roles, PWLLEs shaped core pedagogical choices, influenced language and tone, and challenged normative assumptions embedded in traditional expert-driven educational approaches.^85^ By centering lived experience from the outset, the *CARING in Action* initiative explicitly aimed to address the inequities and stigma that individuals with chronic pain frequently encounter in healthcare communication. As a direct result of their feedback describing experiences of disbelief and stigmatizing language, we revised our training materials to incorporate structured validation prompts, eliminated potentially stigmatizing terminology, and added co-developed communication scripts that emphasize partnership, respect, and shared decision-making. The voices of PWLLEs were prioritized as foundational sources of knowledge, guiding the development of a resource that acknowledges structural marginalization in clinical encounters. This patient-oriented approach was essential to ensuring the acceptability and relevance of the tool for diverse learners and clinical settings.^85^

The process of working across disciplines, geographies, and positionalities presented a series of complex challenges. Differences in conceptual frameworks, professional vocabularies, and institutional timelines required ongoing negotiation, patience, and flexibility. Team members included PhD trainees, healthcare professionals, researchers, knowledge mobilization specialists, and PWLLEs, each navigating competing demands and caregiving responsibilities. Several collaborators were unable to remain engaged throughout the entire process due to personal or professional constraints. PWLLEs were compensated for their time spent on the project, and trainees received awards related to this project, but this work was taking place in addition to their core responsibilities. Coordination was further complicated by geographic dispersion, as team members were based across Canada. To accommodate this reality, the group relied on virtual meetings and asynchronous communication tools. While this enabled flexibility, it also posed limitations in fostering real-time dialogue and relationship-building.

One of the most salient challenges was establishing a shared language not only in terms of spoken or written communication, but epistemologically. Academic imperatives for efficiency, deliverables, and timelines were at times in tension with the slower, relational processes required for meaningful collaboration and consensus-building with PWLLEs. We also recognized that preferences for engagement varied across participants, with some valuing more relational and dialogue-based approaches. Although much of the collaboration occurred online to accommodate geographic dispersion, the team also met in person during two annual Chronic Pain Network meetings, which facilitated relationship-building and more in-depth dialogue. These experiences highlighted the value of combining virtual and in-person engagement strategies when undertaking patient-oriented co-creation initiatives, and we will continue to prioritize flexible, relational, and inclusive engagement approaches as the CARING in Action resource is refined and expanded.

Acknowledging this, the team made intentional efforts to slow down and create space for reflection, iterative dialogue, and relational accountability. Recognizing power dynamics was also central to the collaborative process. Reflexivity practices were embedded throughout the project to critically examine the influence of positionality, privilege, and institutional authority.^86^ Deliberate strategies were implemented to mitigate hierarchical structures and encourage horizontal participation. These included co-facilitated meetings, transparent decision-making mechanisms, and regular feedback loops. Perhaps one of the most enduring insights from this process was the recognition that co-creation extends beyond representation; it requires trust, humility, and a commitment to ongoing relationship-building.^87^ Cultivating an environment of mutual respect and psychological safety proved essential not only to the success of the tool’s initial design but also to its long-term acceptability, scalability, and sustainability. To support sustainability, the CARING in Action modules were developed using Articulate, a widely used e-learning platform that facilitates future content revisions and expansion. The resource will continue to be disseminated through the Chronic Pain Network and associated knowledge mobilization activities. Future updates will be informed by user feedback, emerging evidence, and evolving best practices in pain communication to ensure relevance. These lessons continue to inform the ongoing refinement and dissemination of *CARING in Action* through the Chronic Pain Network and SPOR communities, with a focus on extending co-creation practices to new clinical and educational contexts and deepening their impact on equity and stigma reduction in pain care.

### Equity, diversity, and inclusion reflections

Equity, diversity, inclusion, Indigeneity, and accessibility principles were actively upheld by both academic and PWLLE team members, guiding the design of psychologically safe, respectful, and inclusive collaboration spaces.^88^ Engagement processes included structured dialogue sessions, iterative feedback cycles, and shared decision-making regarding module content and language. At this stage, engagement who self-identify as members of 2SLGBTQ2I+ and Indigenous communities primarily involved consultation with individuals rather than formal partnership with Indigenous communities or governance bodies. While these early consultations informed content development, deeper engagement aligned with Indigenous research principles (e.g., community partnership, relational accountability, and OCAP®^89^ principles of Ownership, Control, Access, and Possession) will be necessary in future phases.

Our training module offers resources in both English and French, increasing accessibility across Canada. We also applied accessibility-informed design principles to the graphics and learning modules. The Articulate platform supports a read-aloud function, and graphics were developed to meet digital and print contrast standards. Additionally, gender and racial representation were intentionally considered in module characters and demonstration videos to reflect the diversity of individuals experiencing pain and to acknowledge the stigma that can shape these healthcare interactions.

## Discussion

### Mobilization implications

The *CARING in Action* framework offers a unique contribution to clinical practice, health professions education, and implementation research by addressing communication, one of the top patient-identified priorities in chronic pain care, alongside trust and stigma. While several educational interventions exist to improve clinician-patient communication, many focus narrowly on generic communication skills or disease-specific protocols, without fully integrating patient perspectives or addressing systemic inequities. Developed through a co-creation process that meaningfully engaged people with lived experience, and utilizing strategic knowledge mobilization tactics, the CARING model provides a concrete approach to improving clinician-patient interactions in settings where pain is often misunderstood and/or minimized.

By adapting principles of empathy, clarity, and respect, the framework equips healthcare professionals across disciplines (such as medicine, nursing, physiotherapy, and allied health) with essential skills for fostering inclusive, compassionate, and trust-building communication in a format designed to be feasible and readily implemented. As a knowledge mobilization intervention, the training module directly addresses long-standing gaps in pain education by embedding real-world patient narratives and equity-informed practices at the core of professional learning. In educational contexts, *CARING in Action* offers a replicable model for integrating lived-experience-informed content into undergraduate, graduate, and continuing education curricula. Its adoption into formal training programs could fill a critical void in pain communication education and create a shift toward participatory pedagogy in health education, distinguishing it from existing communication training approaches by centering equity, lived experience, and practical implementation guidance.

### Implementation reflections

From an implementation science perspective, the CARING *in Action* framework is created with future adoption, integration, and scalability in mind. The training’s multimodal structure enhances its feasibility across a range of academic and clinical environments, including both in-person and virtual settings. The current resource was developed primarily within the context of adult chronic pain care; future work should explore adaptation across the lifespan and within pediatric, adolescent, and older adult pain settings. Early feedback from key interest holders suggests high perceived relevance, emotional resonance, and practical value, all of which are indicators of strong potential acceptability. Anticipated reach includes integration into interprofessional education programs, provincial pain networks, and national continuing professional development platforms.

Dissemination efforts will focus on integrating the resource across the continuum of professional learning, including pre-licensure education, graduate training, and continuing professional development programs across healthcare disciplines. Partnerships with academic institutions, pain networks, and knowledge mobilization organizations will support broader adoption, while future implementation work will evaluate uptake, user experience, and opportunities for ongoing refinement. Although a formal evaluation is forthcoming, the design process has laid the groundwork for assessing key implementation outcomes such as acceptability, feasibility, adoption, reach, and fidelity.^90^ Future steps include piloting the training in diverse Canadian academic and clinical settings, collecting both qualitative and quantitative feedback from learners and organizational interest holders, and refining the module for broader dissemination through national platforms such as the Chronic Pain Network. Ultimately, *CARING in Action* lays the foundation for system-level transformation in how pain is communicated, how patients are engaged, and how clinical empathy is cultivated and sustained across the healthcare continuum. Future implementation and evaluation studies will examine key outcomes, including interest holder engagement, adoption, reach, acceptability, and sustainability across diverse clinical and educational settings. Such work may benefit from implementation-focused frameworks to support the systematic evaluation of these outcomes.

The development of the *CARING in Action* framework and training module illustrates that co-creation is not only a feasible approach to educational tool design but also an ethical and impactful model for bridging science, clinical practice, and lived experience. Based on sustained collaboration, equity-centered principles, and shared decision-making, this initiative generated a pedagogically robust and contextually relevant resource that directly responds to the real-world needs of individuals living with chronic pain. More than a training intervention, *CARING in Action* exemplifies a patient-oriented knowledge mobilization strategy that advances both educational innovation and implementation ethics. It offers a practical case study in how interdisciplinary teams can meaningfully collaborate to translate evidence into accessible, experience-informed solutions for healthcare communication. The online format also supports iterative refinement through future quality improvement cycles informed by user feedback, implementation outcomes, and emerging evidence in pain communication and education. Future phases of the project will focus on evaluating implementation outcomes, including clinical adoption, perceived value, and impact on communication quality. Such efforts may support broader goals of strengthening pain communication competencies within healthcare education and pain care practice. Finally, we believe that this modest but pivotal step toward better education will foster more empathetic conversations around pain, improve care, and ultimately reduce the costs of chronic pain management and improve health outcomes for Canadians.

## Supplementary Material

SupplementalMaterial_CARING.pdf
