# Supplementary material for: “CARING in Action”: A Knowledge Mobilization Project on Co-Creating Evidence-Based Communication Training in Chronic Pain Care for Healthcare Professionals
Source: Can J Pain. 2026 Aug 6;10(1):2696249. doi: 10.1080/24740527.2026.2696249 (PMC13449722; doi:10.1080/24740527.2026.2696249)
Supplement: Supplementary file 1 [file UCJP_A_2696249_SM6317.pdf]

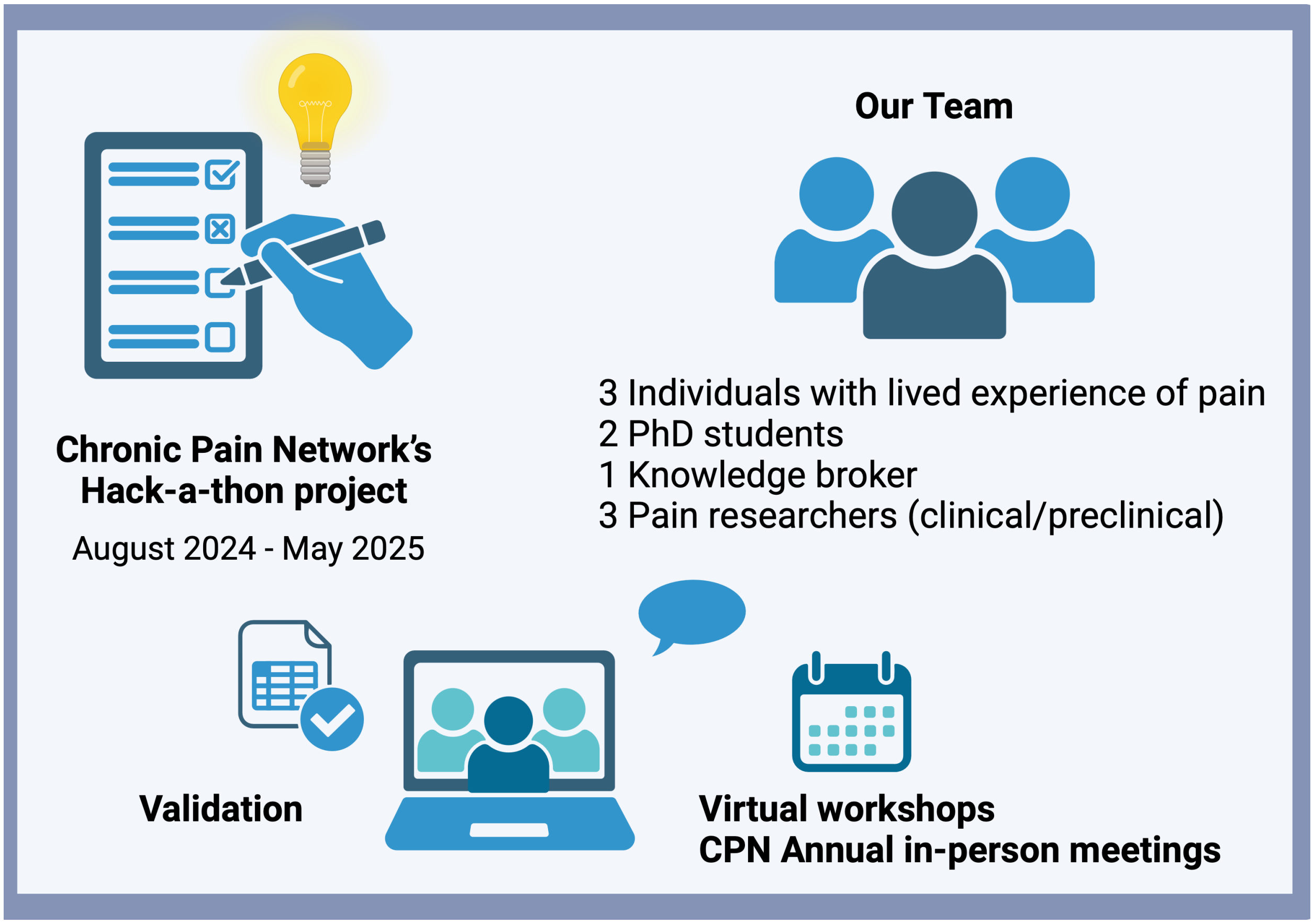

Supplemental Figure 1. CARING methodology and workflow.

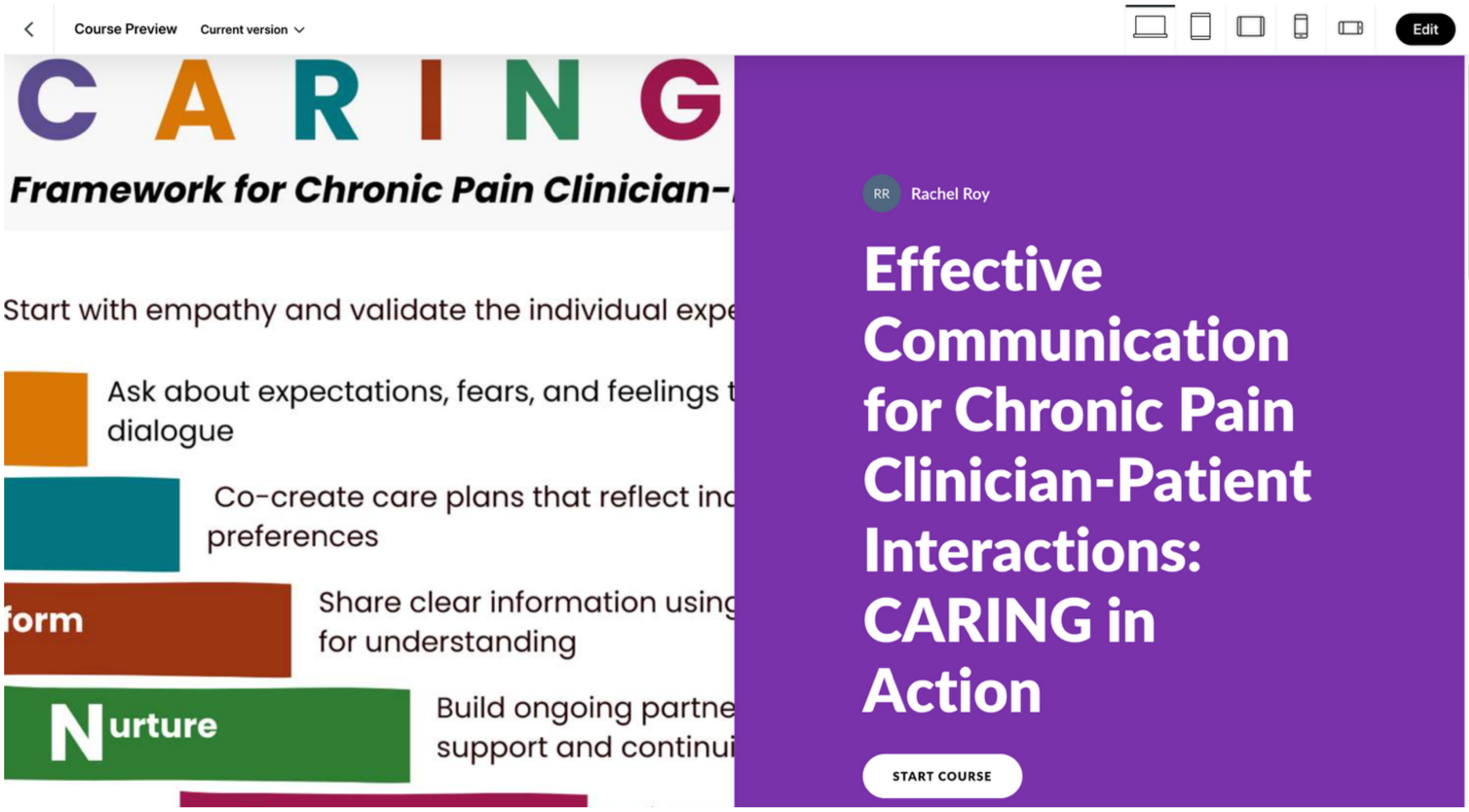

Supplemental Figure 2. CARING training course start page.

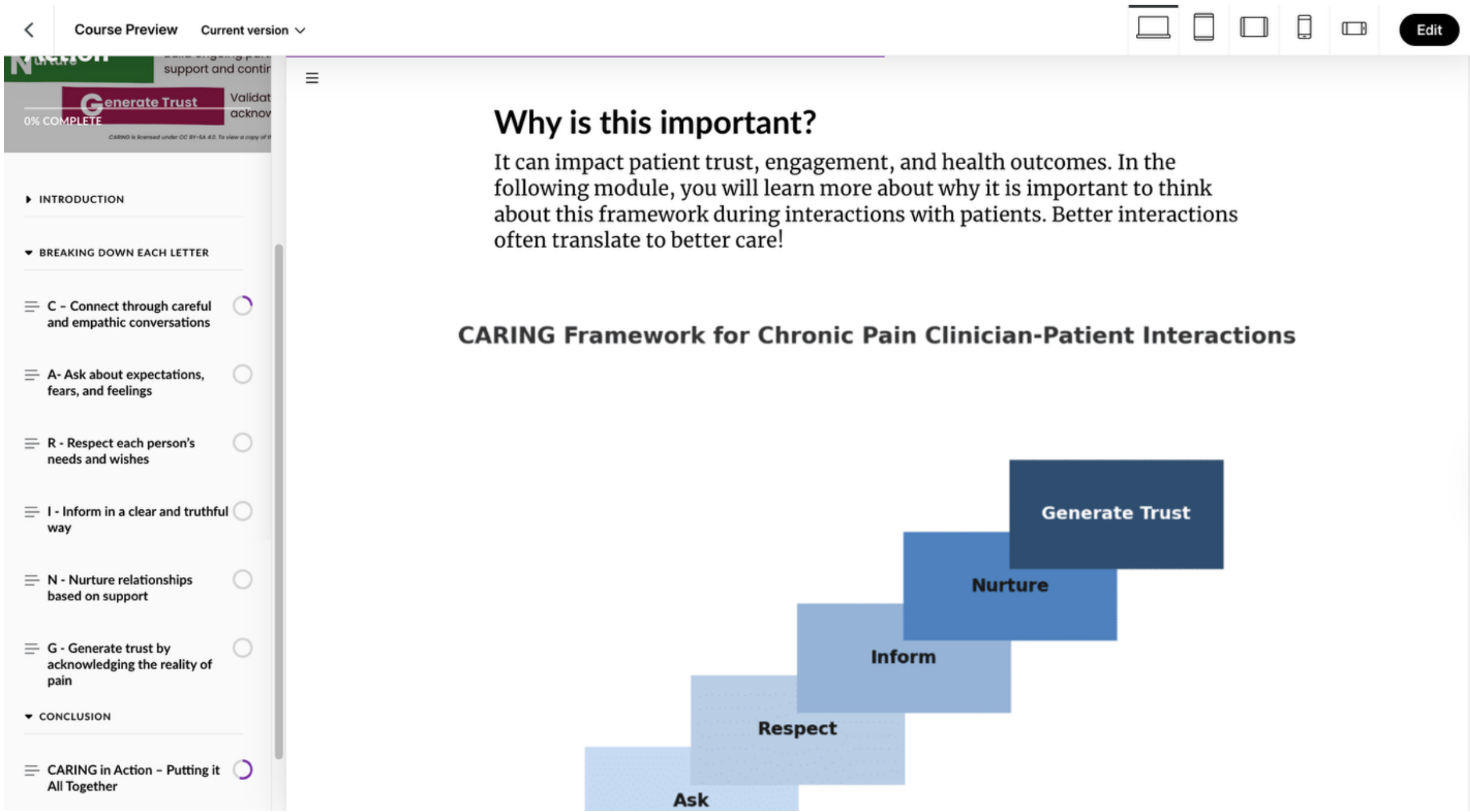

Supplemental Figure 3. CARING training course.

Supplemental Table 1: Guidance for Reporting Involvement of Patients and the Public (GRIPP2)

**Section**	**Item**	**Reported on page No**
Aim	Report the aim of PPI in the study	Abstract page 2 and main text pages 7-8.
Methods	Provide a clear description of the methods used for PPIE in the study	Pages 10-14.
Study results	Outcomes – Report the results of PPI in the study, including both positive and negative outcomes	Pages 14-20.
Discussion and conclusions	Outcomes – Comment on the extent to which PPI influenced the study overall. Describe positive and negative effects	Pages 20-23.
Reflections/critical perspective	Comment critically on the study, reflecting on the things that went well and those that didn't, so others can learn from this experience	Page 17-20 and 21-23.

PPI=patient and public involvement
